# An Evaluation of the Impact Effect on the Surface Microstructure and Its Induced Temperature Changes during Ultrasonic-Assisted Micro-Forging

**DOI:** 10.3390/ma17164123

**Published:** 2024-08-20

**Authors:** Zidong Yin, Weiqiang Wan, Ming Yang

**Affiliations:** 1 Graduate School of System Design, Tokyo Metropolitan University, 6-6, Asahigaoka, Hino-shi 191-0065, Japan; 2 School of Mechanical Engineering and Electronic Information, China University of Geosciences, Wuhan 430074, China; wanweiqiang@cug.edu.cn

**Keywords:** ultrasonic vibration-assisting, microstructure evolution, acoustic softening, impact effect, micro-forming

## Abstract

In the field of ultrasonic-assisted micro-forming, in addition to acoustic softening, impact effects also play a significant role, especially in terms of influencing the deformation behavior of surfaces, such as by generating more deformation on surface asperity. In this study, to understand the mechanisms involved in the effect of an impact, ultrasonic-assisted micro-forging tests were conducted on commercially pure copper, pure aluminum, and pure titanium. A method that can measure the increment in the temperature during ultrasonic vibration was developed. As a result, changes in the surface temperature of the material under the impact effect and acoustic softening were measured. It is indicated that, during ultrasonic vibration, the heat generated through acoustic softening is very limited and the main heat increase occurs after the impact effect. Once the impact effect occurs, the surface temperature increases with increasing amplitude. Nevertheless, for materials with different crystal structures, the influences of the impact effect are also different. The surfaces of copper and aluminum soften, creating more surface deformation, but the exact opposite effect is seen on a titanium surface. Observing the evolution of the microstructure on the material surface with EBSD demonstrates that the impact effect on FCC materials can reach deeper below the surface in terms of temperature diffusion compared to titanium. Meanwhile, the impact effect in the case of titanium causes the regeneration of twinning, which is reduced under the influence of the acoustic softening effect, consequently resulting in strain hardening.

## 1. Introduction

Since Blaha and Langenecker [1] applied ultrasonic vibrations in zinc bar tensile test experiments and found that ultrasonic vibrations can effectively reduce the forming force, the so-called Balaha effect has been extensively studied by researchers in both industrial and academic fields. The application of ultrasonic vibration-assisted methods to other plastic-forming processes, such as tension [2], drawing [3,4,5], and compression [6], has been proven to be quite an effective approach compared to other high-density energy-assisted forming processes, like resistance heating [7] and the use of lasers [8]; the use of ultrasonic vibrations not only has lower costs and higher efficiency but could also be more effective. In addition, due to the increasing demand for micro-parts seen in recent years, micro-forming technology is now considered to be a promising processing method that can satisfy the mass production of miniature parts [9]. However, as the geometric dimensions of materials gradually decrease from the macro-scale to micro-scales, the size effect becomes increasingly significant and difficult to ignore. M.W. Fu reported that the size effect results in negative alterations, such as surface roughness, increased forming stresses, and decreased forming accuracy in the process behavior, even when the main geometrical features remain constant [10]. Naturally, researchers have once again applied ultrasonic energy to micro-forming processes, such as micro-extrusion [11], micro-deep drawing [12], and micro-bending [13], and have discovered that ultrasonic vibrations could also offer beneficial effects in the case of micro-forming, including the surface effect, which improves surface quality [14], and the volume effect, which reduces stresses [15]. Furthermore, research into the mechanisms by which ultrasonic energy improves the quality of micro-forming has also received considerable attention in the last decade.

In general, acoustic softening is considered to be a major reason for the enhancement of plastic deformation within a certain volume range. According to the explanation of this theory, during ultrasonic vibration processes, dislocations will absorb the ultrasonic energy. This reduces the activation energy needed for dislocations to overcome lattice obstacles, thereby increasing their mobility. Consequently, this leads to a macro-level reduction in stress [16]. It is suggested that stress reduction due to acoustic softening would be intensified when the ultrasonic amplitude rises [17]. In addition to acoustic softening, a residual hardening effect was observed during an ultrasonic vibration-assisted aluminum compression test; in that study, this residual hardening effect was linked to the increased multiplication of dislocation density [18]. Acoustic residual softening was also observed when ultrasonic vibration was superimposed, which was more closely related to ultrasonic duration than ultrasonic amplitude [19]. For HCP materials, the influence of the acoustic softening effect on the Hall–Petch behavior was observed during ultrasonic vibration-assisted micro-strain tests of pure titanium foils. The decrease in the Hall–Petch slope due to ultrasonic vibrations became more pronounced with increasing strain [20].

Furthermore, when focusing on material surfaces, in addition to acoustic softening, the effect of an impact also plays a more significant role on the surface deformation behavior of a material [21]. The impact effects occur because a vibrating punch can cause contact to be lost with the workpiece surface during the process, which can also be distinguished from acoustic softening via the use of dynamic force waveform analysis [22]. It has also been demonstrated that the impact effect can create deeper plastic deformation layers [23] or be used in micro-forming treatments for foil materials [24]; the enhancement effect is directly proportional to the vibration amplitude. However, most of the existing studies on the impact effect induced by ultrasonics in micro-forming only provided observational results, leaving the underlying mechanisms relating to the impact effect largely unexplored. One of the controversial factors involved is the temperature change during ultrasonic-assisted processing. In an ultrasonic-assisted micro-drawing experiment, B. Meng observed that the local temperature near the position of the drawing die was notably intensified in the process [25]. On the other hand, Liu found minimal overall temperature changes when conducting compression tests on titanium samples [26]. Due to the fact that the temperature during vibration processing is difficult to measure, and especially the real-time changes in the surface temperature, it is often overlooked in most ultrasonic-related studies. This neglect of a critical factor affecting the impact effect has led to misunderstandings about its characteristics in relation to surface deformation. Therefore, it is worthwhile to investigate the temperature change on the material’s surface to help understand the underlying mechanisms of the impact effect.

The aim of this study was to investigate the temperature change on the material surface under acoustic softening and impact effect during ultrasonic-assisted micro-forging and to better understand the mechanism of the impact effect. A method that can measure the increment of the temperature during ultrasonic vibration was developed. Furthermore, to understand the impact effect on materials with different temperature dependence, strain rate dependence, and microstructure, ultrasonic-assisted micro-forging was conducted on commercially pure copper, pure aluminum, and pure titanium. In addition, the EBSD (electron backscatter diffraction) technique was adopted to investigate the microstructure evolution after micro-forging and AFM (atomic force microscope) had been used to observe the surface topography. As a result, after comparing the temperature change history and the evolution of the microstructure under the influence of acoustic softening and impact effect in the three materials, the mechanism of the impact effect was discussed.

## 2. Experiment Set-Up and Materials

### 2.1. Design of the Micro-Forging Device

#### 2.1.1. Ultrasonic-Assisted Micro-Forging Test System

**Figure 1:**
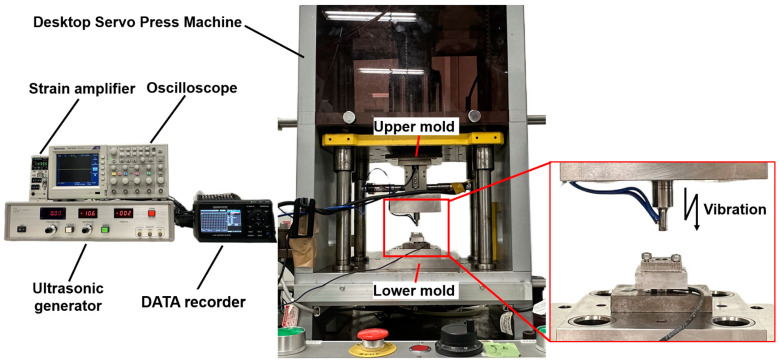
Configuration of the ultrasonic-assisted micro-forging system.

A novel ultrasonic-assisted micro-forging system was used, composed of an upper-mold ultrasonic transducer assembly, a lower-mold dynamic force measurement system, and a temperature monitoring system, as shown in Figure 1. Furthermore, this comprehensive system was set in a Desktop Servo Press Machine (DT-3AW, manufactured by Micro Fabrication Laboratory), which had been specifically engineered to offer deformation control at the micrometer scale (also shown in Figure 1).

#### 2.1.2. Upper Mold of the Ultrasonic-Assisted Micro-Forging System

**Figure 2:**
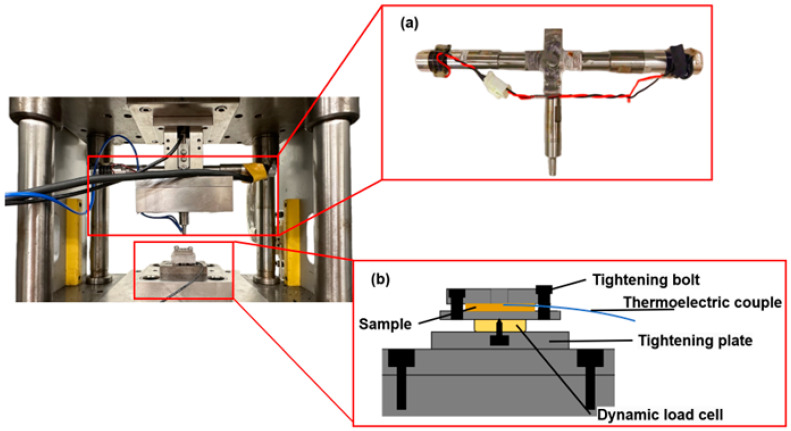
(**a**) Integrated structure of the ultrasonic transducer. (**b**) Schematic of the dynamic force test system.

The primary function of the upper mold is to secure the ultrasonic transducer, which is connected to a load cell via a slider block. Figure 2a shows the integrated structure of the ultrasonic transducer. Two horizontal transducers generated ultrasonic vibration, originally in the same phase. Then, the punch received the vibration, which was amplified and transformed in a vertical direction by a specially designed horn. Finally, the maximum amplitude of 6 µm on the punch tip was measured by a laser displacement meter (LC-2400, KEYENCE, Tokyo, Japan). The input electrical signal for the ultrasonic transducers was a sine wave, with a frequency of 60 kHz, produced by an ultrasonic generator.

#### 2.1.3. Lower Mold of the Ultrasonic-Assisted Micro-Forging System

To measure the rapid and minute changes in stress during the vibration process, a dynamic force measurement system consisting of a dynamic load cell (Kistler 9132B, Winterthur, Switzerland), oscilloscope (Tektronix, DPO2014, Beaverton, OR, USA), and data recorder (OMRON ZR-RX70, Kyoto, Japan) was set up, as shown in Figure 2b. Because the dynamic load cell (Kistler 9132B) has high-frequency responsibility, it was suitable for measuring a rapidly changing force, and any tiny change in force would be detected. The presence or absence of the impact effect could be monitored in real time during the experiment using the display of the connected oscilloscope.

**Figure 3:**
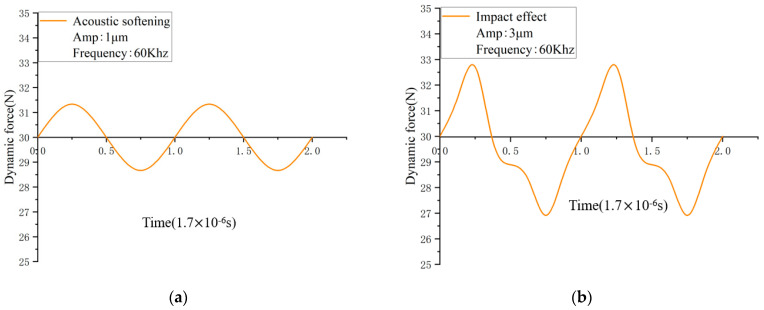
Waveform of (**a**) acoustic softening, (**b**) impact effect.

According to the definition, the impact effect is generated by the separation between the punch and the specimen’s surface. Hence, when the waveform displayed on the oscilloscope was a sine wave, this indicated continuous contact between the vibrated punch and the specimens, as in Figure 3a. It also meant that only acoustic softening was taking place at that moment; otherwise, the waveform would be distorted to a non-sine waveform, as shown in Figure 3b, which indicated that the impact effect had happened. In this study, a sine waveform would occur at the amplitudes of 0.5 μm and 1 μm. Then, it would be distorted to a non-sine waveform when the amplitude increased to 2 μm, 3 μm, and 3.5 μm. The reliability of this method was demonstrated in the previous study [21], and it performed well in the later experiment.

#### 2.1.4. Surface Temperature Monitoring System and the Specimen

**Figure 4:**
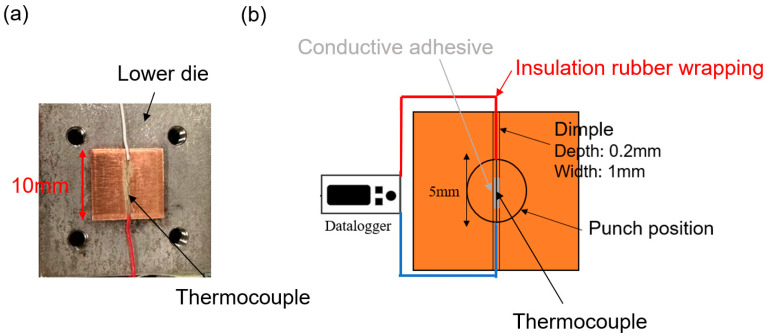
(**a**) Specimen on the lower die. (**b**) Schematic of the surface temperature monitoring system.

Obtaining accurate temperature change data during the experiment was challenging. Firstly, throughout the experimental process, the punch kept making contact with the specimen surface, since the use of non-contact temperature measurement devices is precluded. Meanwhile, the contact temperature sensor made it hard to measure the surface temperature without interfering with the vibrations. Thus, to obtain the surface temperature change during the process correctly, a skinny thermoelectric couple of 0.1 mm diameter was used. It was placed on the sample surface, where a tiny dimple with a depth of 0.2 mm and a width of 1 mm was machined while processing. To ensure that the vibration would not be disturbed by the thermoelectric coupling during processing, it could be secured at the bottom of the dimple using a conductive adhesive, as shown in Figure 4.

**Figure 5:**
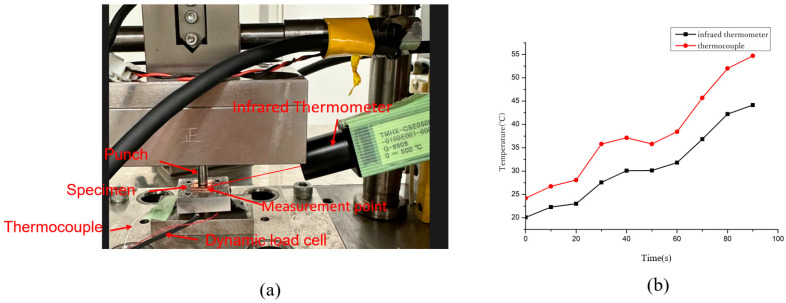
(**a**) Calibration of temperature by infrared thermometer. (**b**) Temperature change curves with the two methods.

Meanwhile, to verify the accuracy of the temperature monitoring system, non-contact infrared thermometers were used to check the temperature measured by the thermocouples, as shown in Figure 5a. However, the infrared thermometer cannot directly measure the area where the punch and the specimen are in contact, which is also the reason why thermocouples were selected for the formal experiment, and the measurement point could only be as close to the contact area as was possible. During the testing process, a thermocouple was placed in a dimple. When the ultrasonic generator was activated and the punch began to vibrate vertically, the temperature change was measured by both an infrared thermometer and the thermocouple, as shown in Figure 5a. The results of the temperature change curves are shown in Figure 5b, which shows the trend of the temperature throughout 900 ms, and to eliminate noise and errors in the process, the temperature changes were averaged every 100 ms, thus fitting a spline curve in which the red line represents the temperature measured by the thermocouple and the black line represents the temperature measured by the infrared thermometer.

### 2.2. Forging Test Conditions and Materials

All ultrasonic-assisted micro-forging experiments were carried out at room temperature (23 °C) using the equipment described earlier. In order to evaluate the impact effect on materials with different temperature dependencies and crystalline structures, specimens of three materials were selected for testing. Each specimen was manufactured to a size of 10 mm × 10 mm with a thickness of 2 mm. Since each sample was taken from the larger piece of its respective material, the physical properties among different samples of the same material could be considered constant, as shown in Table 1.

During the experiments, different amplitudes would influence the surface deformation quantities. Therefore, to investigate the influence of strain rate, amplitudes of 0.5, 1, 2, 3, and 3.5 μm were applied to the specimens, respectively, and in the current study, the amplitude was defined as the displacement between the punches from the equilibrium position to the position of maximum movement.

The preload is one of the factors influencing the presence or absence of the impact effect. According to the conclusions drawn by [24], under identical vibration conditions, materials with different elastic moduli exhibit different critical amplitudes for the transition from acoustic softening to the impact effect. The impact effect can be observed more rapidly under lower preloads. Therefore, to ensure that the impact effect and acoustic softening could be observed for each material, a consistent preload of 30 N has been applied in this study. 

Each processing lasted for 60 s to ensure temperature stabilization. The influence of frequency was not considered in this study, so the ultrasonic generator produces a constant vibration frequency of 60 ± 2 kHz.

## 3. Results and Discussion

### 3.1. Evaluation of the Temperature Change under Different Amplitudes

**Figure 6:**
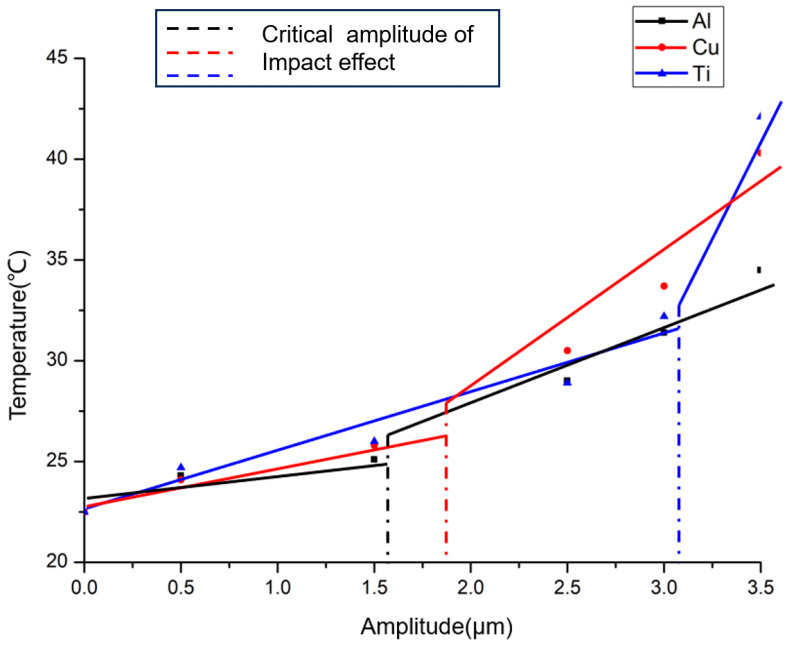
Maximum temperature under different amplitudes.

Based on the temperature monitoring system, the maximum temperature of the surface of the specimen during 60 s of vibration at different amplitudes was plotted and, to better compare the acoustic softening and impact effects, piecewise lines were used, with the first line showing the trend in the maximum temperature of the surface during acoustic softening, and the second showing the trend in the maximum temperature of the surface under the impact effect, as shown in Figure 6. Under the same preload, the critical amplitudes at which the impact effect occurred differed due to the influence of their respective elastic moduli: aluminum has a critical amplitude of 1.6 µm, copper of 1.8 µm, and titanium of around 3.1 µm. However, it is evident that after the impact effect had taken place, the rate of temperature increase became much more pronounced and rapid compared to the low-amplitude phase with only acoustic softening. In the initial phase, when the amplitude was below the critical point, only acoustic softening took place. This also implied that during the whole vibration process in this phase, the punch kept in constant contact with the material surface, and the specimens only underwent elastic deformation. Thus, the temperature change for each material was fairly minimal. For aluminum, when the amplitude was increased from 0.5 μm to 1.5 μm, the surface temperature obtained was still around 25 °C, with a change of about 2 °C. At this stage, the copper was also similar due to its microstructure. Even for titanium, the temperature rise was only about 5 °C when the amplitude increased from 0.5 μm to 3.0 μm. Once the amplitude exceeded the critical amplitude necessary for the impact effect, the surface of the material began to undergo plastic deformation during the vibration. Consequently, from this point on, the amplitude’s influence on temperature intensified to different degrees. For FCC materials like copper and aluminum, under the influence of the impact effect, even though the amplitude only increased by 0.5 μm (from 2.5 μm to 3 μm), the temperature rose nearly 3 °C. This increment was already equivalent to the maximum rise observed before the appearance of the impact effect. As the amplitude increased by a further 0.5 µm, Al specimens reached their maximum temperature of 35 °C, while copper exceeded 40 °C. For the HCP material, titanium, although under the pre-load conditions of this time experiment the critical amplitude for the impact effect was relatively high, about 3.5 μm, the results demonstrated a pronounced sensitivity of the titanium surface temperature to the impact effect. Once it occurred, an amplitude increase of less than 0.5 μm could result in a temperature increase of more than 10 °C compared to before the impact effect occurred.

During ultrasonic vibration, as the amplitude increases, the mechanism of ultrasonic vibration on the deformation of the metal material undergoes a transformation [21]. At minimal amplitudes, only a tiny amount of deformation was induced by the vibration, and the ultrasonic energy was absorbed by the material’s elastic deformation in the form of acoustic softening. This was utilized to enhance the internal defects in the metal material, such as void defects and dislocations. During this phase, more dislocating motions would be created and repeated friction would be caused under the influence of ultrasonic vibrations in a short time, resulting in heat generation. Nevertheless, this kind of generated heat was remarkably limited. As shown in Figure 6, the maximum temperature rise was limited to about 2 °C. When the amplitude exceeds the critical point, the impact effect begins to play a dominant role. Therefore, some of the asperities on the material’s surface pass from elastic deformation to plastic deformation, and as the amplitude increases, so does the extent of the plastic deformation. Typically, the temperature increase caused by the self-heating effect of materials is more easily observed at higher strain rates. At a frequency of 60 kHz, an increase in amplitude means greater deformation, leading to increased heat generation due to the self-heating effect and consequently a temperature rise. As shown in Figure 6, once the amplitude surpasses the critical amplitude, the rate of temperature increase became more drastic with increasing amplitude. 

According to A. Bragov [27], for the same strain rate, the temperature increase due to the self-heating of copper is greater than that of aluminum, and a higher strain means a greater temperature change, which was consistent with the trends shown in Figure 6. In addition to the heat generated by self-heating, as the deformation increased, the frictional resistance between the specimen and punch surface that the ultrasonic energy must overcome also increased. This led to a greater proportion of the ultrasonic energy being converted into heat through friction. However, the relationship between the friction coefficient and ultrasonic energy was not considered in this paper.

**Figure 7:**
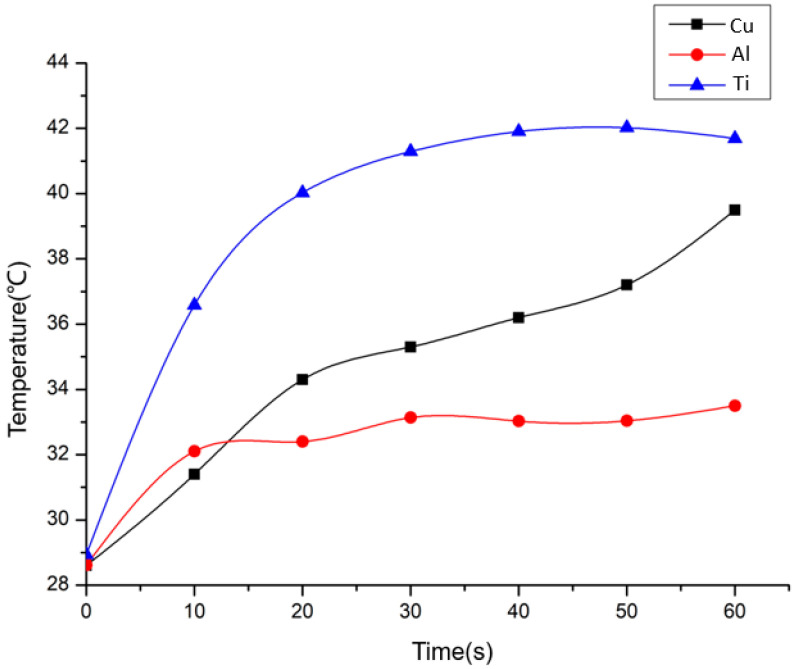
Temperature history within 60 s at 3.5 μm amplitudes.

To further understand the underlying reason for the different temperature changes induced by the impact effect on HCP and FCC samples, an investigation of the temperature history within 60 s at 3.5 μm amplitudes was presented, and the spline curve was fitted to the temperature values for every 10 seconds per minute, where to minimize noise and error the temperature value at each point was the average of the temperature change over the 10 seconds, as shown in Figure 7. Notably, both copper and aluminum demonstrated a relatively uniform rate of temperature increase over the 60 s duration, progressively rising from their initial temperatures to their peak values. Meanwhile, the temperature increase in titanium was predominantly concentrated within the first 30 s, after which the increase stopped and even began to show a decrease.

The different temperature change trends observed for the three materials could likely be attributed to their different thermal conductivities. For both copper and aluminum, the temperature increase resulting from strain during each impact was rapidly diffused due to their relatively high thermal conductivity. In addition, the lower hardness of aluminum compared to copper meant that the strain ended earlier, which also led to a faster peak temperature being reached. On the other hand, titanium’s extremely low thermal conductivity meant that heat could not be effectively transferred to the deeper regions of the material from the beginning of the strain. As a result, there was a rapid accumulation of heat in the initial twenty seconds of deformation.

Notably, since this experiment was not conducted in an adiabatic system, the observed temperature increases have already been considered in the heat loss. However, due to the uniform thickness and dimensions of the samples, as well as the minimal deformation during the process, the heat loss could be simplified as being proportional to the thermal conductivity of the material.

### 3.2. Effect of Temperature on Surface Topography

**Figure 8:**
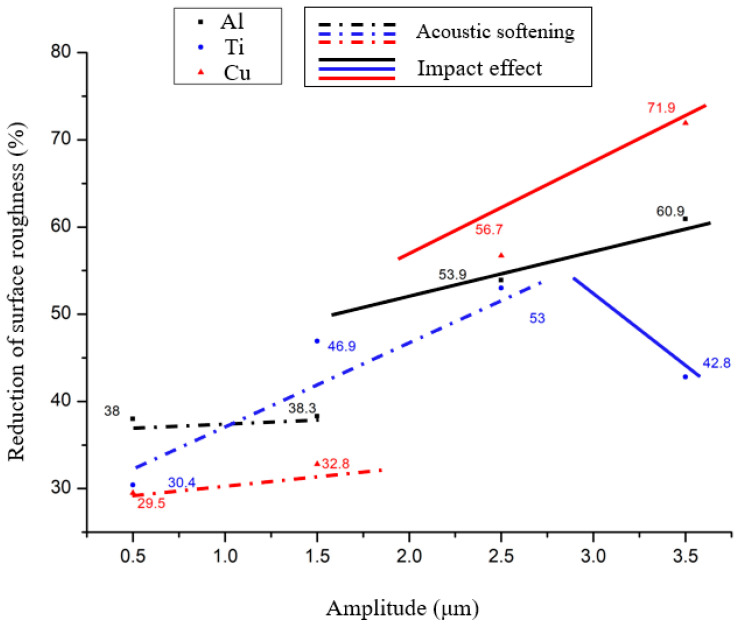
Reduction in surface roughness at different amplitudes.

To clarify the influence of temperature rise induced by ultrasonic vibrations on the deformation behavior of material surfaces, reductions in the surface roughness and surface topography of the materials were investigated using Atomic Force Microscopy (VN-8010, Keyence, Tokyo, Japan). Figure 8 shows the decrease in surface roughness of the central area of the material surface within 200 × 200 μm^2^ after vibration at different amplitudes. The reduction in surface roughness $∆R_{a}$ (μm) could be defined by the following equation:

(1) $$∆R_{a}=\frac{R_{raw}-R_{a}}{R_{raw}}\times 100\%$$

where R_raw_ and R_a_ are the original surface roughness and that after ultrasonic vibration, respectively. It can be seen that all three materials exhibited different degrees of reduction in surface roughness compared to their initial values during the acoustic softening phase. In particular, titanium showed the most significant reduction, approaching nearly 20%. In contrast, copper and aluminum showed only a marginal reduction in surface roughness, of around 1% to 2% throughout this phase, with increasing amplitudes. However, upon entering the impact effect stage, the roughness of the aluminum and copper surfaces immediately transitioned to a new level, showing significant reductions of 14% and 15.5%, respectively. Furthermore, with a further increase in amplitude by one micrometer, the degree of reduction intensified, reaching 13% for aluminum and 8% for copper. Surprisingly, titanium showed an opposing trend, with a 9% decrease in the reduction in surface roughness following the onset of the impact effect.

For FCC materials aluminum and copper, the reduction in surface roughness was closely correlated with temperature. During the acoustic softening stage, only elastic deformation occurs, and hence the majority of the reduction in surface roughness could be attributed to the pre-load. Although an increase in amplitude also caused minor temperature fluctuations, the magnitude of this temperature rise just affected some extremely tiny surface asperities. Therefore, during this phase, the decrease in roughness was not so obvious. As temperatures rose rapidly under the influence of the impact effect, the thermal softening effect induced by the temperature began to influence the materials. During the dynamic compression of copper and aluminum, the increase in temperature resulted in a decrease in the true stress required for deformation, as was concluded by Samant [28]; additionally, it was also noted that for the same strain rate, the stress reduction in copper exceeds that of aluminum for each degree of temperature increase. Thus, as can be seen in Figure 8, the rate of reduction in surface roughness for copper surpassed that of aluminum after the impact effect began. It is worth noting that, although the temperature measured in this experiment was already very close to the surface, it still represents an average value near the surface rather than the instantaneous value at the point of contact between the punch and the test specimen. Therefore, the instantaneous surface temperature is likely to be even higher.

**Figure 9:**
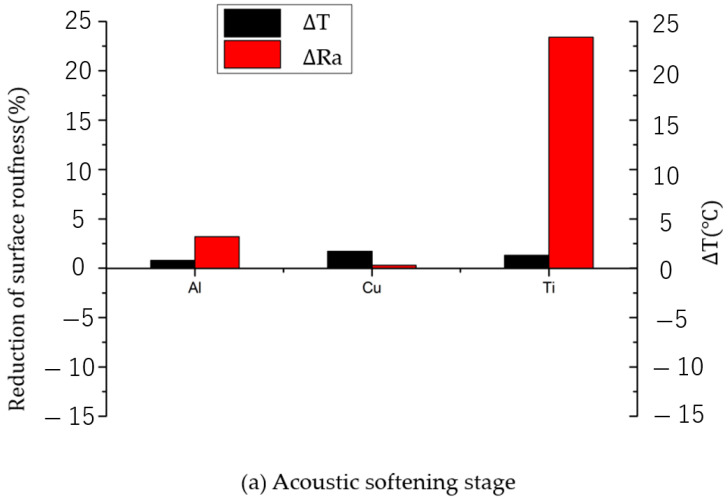

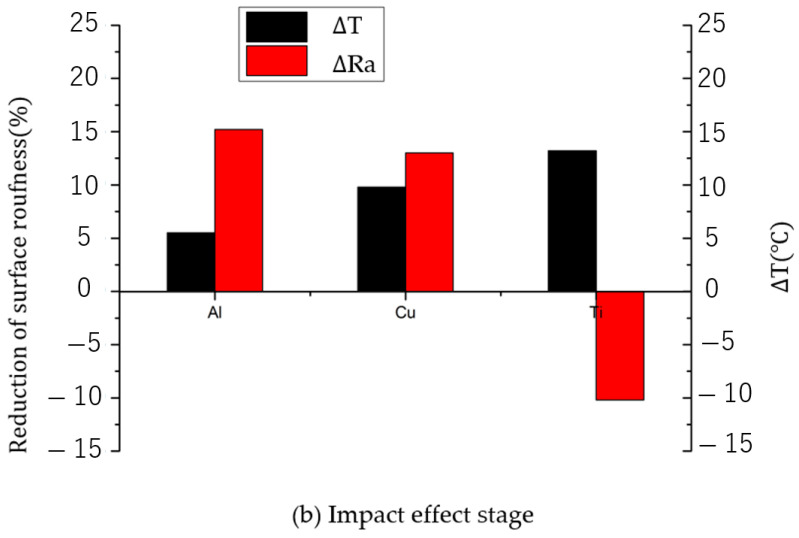
Comparison between temperature increments and reductions in surface roughness at different stages: (**a**) acoustic softening stage and (**b**) impact effect stage.

For titanium, the enhancement of its surface deformation under the influence of acoustic softening was probably due to the ultrasonic energy providing additional energy to break local bonds and distorted grain boundaries in the material. Compared to materials with an FCC microstructure, the crystalline structure of titanium appeared to exhibit a higher absorption rate for ultrasonic energy, especially in cases where the temperature effect was not pronounced, as shown in Figure 9a. Its influence on the Hall–Petch behavior in pure titanium was also proposed by Xin Wei Wang [20]. Ultimately, this resulted in a decrease in yield stress, which was also a common conjecture regarding the acoustoplasticity proposed by Langenecker [1], allowing surface asperities, which originally remained undeformed under the preload, to undergo minor deformation.

As shown in Figure 9b, during the impact effect stage, although the temperature increase on the surface of titanium was observed to be higher than that of copper and aluminum, considering titanium’s higher melting point, the degree of surface softening was not necessarily greater than that of copper and aluminum, which had lower surface temperature increases. Therefore, the reduction in surface roughness of titanium decreases with a temperature increase. An explanation for this phenomenon is that while the temperature increase causes thermal softening, the softening is not sufficient to overcome the work hardening induced by the impact effects, leading to a rebound in surface roughness when the impact effect occurs. For FCC materials like copper and aluminum, it was evident that the increase in temperature results in more pronounced surface softening, and therefore a more pronounced reduction in surface roughness. Although aluminum has a lower melting point than both copper and titanium, its softer material properties mean that the same amplitude creates a greater strain rate, which is also highly dependent on the material properties. Therefore, aluminum could exhibit surface softening even at lower temperature increases. 

**Figure 10:**
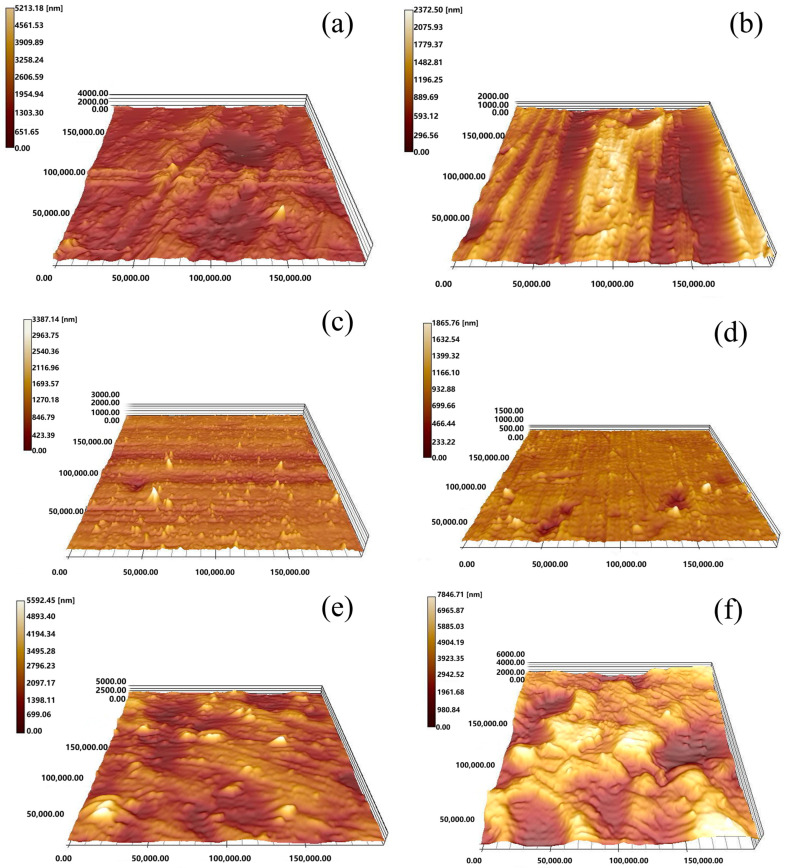
Three-dimensional surface topographies of the surface of Al (**a**,**b**), Cu (**c**,**d**), and Ti (**e**,**f**) with different ultrasonic amplitudes (**a**,**c**,**e**) of 1.5 μm and (**b**,**d**,**f**) of 3.5 μm.

AFM (Atomic Force Microscopy) images provided a more intuitive representation of the influence of different ultrasonic stages on the material’s surface deformation behaviors, as shown in Figure 10. Figure 10a,c,e show the surface morphology of aluminum, copper, and titanium after ultrasonic vibration processing at an amplitude of 1.5 µm, while Figure 10b,d,f depict the surface after processing with a maximum amplitude of 3.5 µm. A comparison showed that the maximum height of asperity on the surface of copper and aluminum decreased from 5213.10 nm to 2370.5 nm and from 3387.14 nm to 1865.76 nm, respectively, when the mechanism of ultrasonic vibration transitioned from acoustic softening to the impact effect. In contrast, titanium behaves differently: under the influence of the impact effect, its maximum surface asperity’s height increased from 5592.45 nm under acoustic softening to 7846.71 nm. The changes in surface morphology also showed a positive correlation with the changes in roughness and temperature discussed above.

### 3.3. The Microstructure Analysis

**Figure 11:**
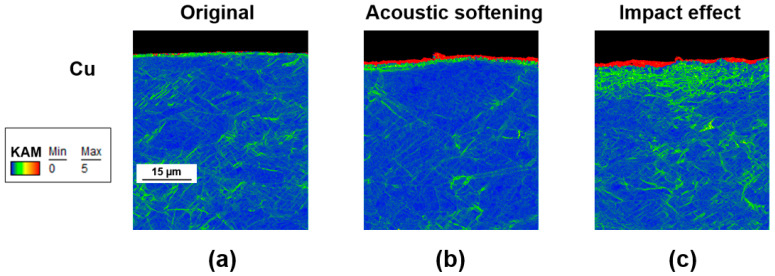
Kernel average misorientation (KAM) maps (in °) of copper with (**a**) original, (**b**) 1.5 μm, and (**c**) 3.5 μm.

In order to comprehensively understand the mechanism of the impact effect and the influence of it on FCC and HCP material, microstructural observations of EBSD (Electron Back Scattered Diffraction) of copper and titanium are provided. Figure 11 shows the KAM (Kernel Average Misorientation) distribution of copper processed at different amplitudes. As can be seen, after being applied an amplitude of 1 µm, the copper surface generated significant deformation to a depth of about 2 micrometers, as shown in Figure 11b. However, in the deeper area over a 2-micrometer depth, the dislocation density decreased compared to the original materials. This reduction might be attributed to dynamic recovery induced by acoustic softening. Ultrasonic vibrations provide additional energy, which helps dislocations to overcome lattice boundary resistance, facilitating further slipping. As the impact effect became more dominant, it was clear that more dislocations occurred not only in the region affected by the 3.5 µm amplitude, but also beyond a depth of 5 µm. Deformation could also be seen deeper inside, which was probably due to the increase in temperature leading to thermal softening. Indeed, the distribution of dislocation density followed the pattern of temperature diffusion; the closer to the surface, the higher the temperature, resulting in more concentrated dislocation. As the temperature diffused deeper into the inner material, the dislocation gradually spread out. The influence brought about by temperature could even be observed down to about 50 µm below the material surface, as can be seen throughout Figure 11c, while acoustic softening was more concentrated within the top 20 µm of the surface.

**Figure 12:**
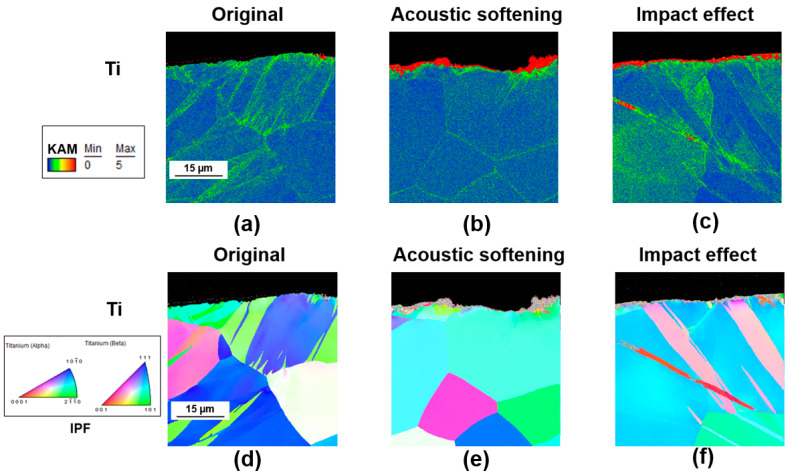
Kernel average misorientation (KAM) maps (in °) and grain orientation maps of titanium with (**a**,**d**) original, (**b**,**e**) 1.5 μm, and (**c**,**f**) 3.5 μm.

The unique crystalline structure of titanium resulted in different influences on acoustic softening and impact effects. Although both the 1.5 µm and 3.5 µm amplitude titanium samples showed slight surface deformation due to ultrasonic vibration, as shown in Figure 12b,c, in contrast to copper, the depth of surface deformation did not increase after the impact effect; almost all the KAM values over 5° were still concentrated on the surface at 2 µm. Figure 12d shows that, in the original material, deformation twinning was more dominant. However, under the influence of ultrasonic softening, it is evident that the amount of deformation twinning was significantly reduced. As a result, the deformation mechanism was transformed from deformation twinning to slippage. In Figure 12b, the dislocation density in the deeper material of the titanium was lower and the deformation became easier, compared to Figure 12a. This was because the reduction in deformation twinning could improve work hardening by reducing the dislocation friction, as reported by Genki Tsukamoto’s 2022 study [29], and the influence of acoustic softening on the work hardening to titanium was directly proportional to the ultrasonic energy density, which was reported by X. Wang [20]. With the impact effect becoming more significant at larger amplitudes, the accompanying high strain rate led to the generation of deformation twinning, as shown in Figure 12f. Within a range of 20 μm below the surface, deformation twinning reappeared; this deformation twinning would induce work hardening again, and when the increase in strain rate reached its maximum at 3.5 μm amplitude, the twinning mechanism was more likely to be triggered [26]. In contrast to the slippage system, the deformation twins became dominant, and the dislocation was difficult. The dislocation density was mainly located along the grain boundaries at a depth of around 20 μm below the surface, as shown in Figure 12c, increasing the lattice friction and making surface deformation more difficult. This also explains why the surface roughness of titanium increased after the onset of the impact effect.

**Figure 13:**
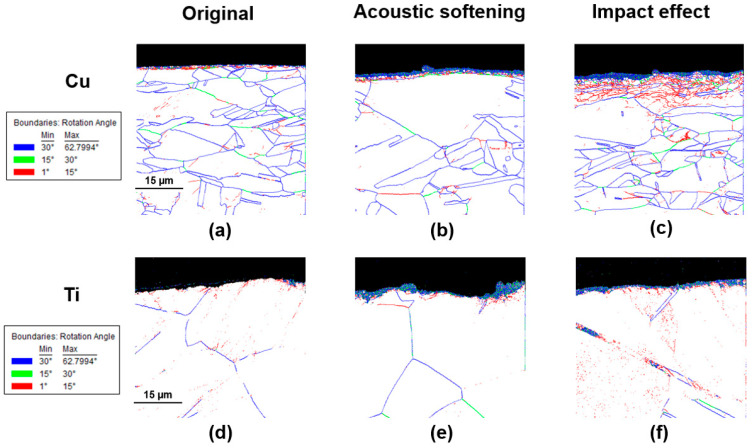
Image quality (IQ) maps with high-end and low-angle grain boundaries of copper and titanium with (**a**,**d**) original, (**b**,**e**) 1.5 μm, and (**c**,**f**) 3.5 μm.

Image quality (IQ) maps with high- and low-angle grain boundaries could further explain why the impact effect led to different results in materials with different crystal structures. Figure 13a–c show that after the impact effect took place, more low-angle grain boundaries were created and piled up under the material’s surface layer in the area directly vibrated by the punch, as compared to before the impact effect, as shown in Figure 13b. Additionally, the distribution of these low-angle grain boundaries also maintained the same trend in temperature diffusion, as shown in Figure 13c. It was these accumulated low-angle grain boundaries that made dislocation movements between the grains of the copper surface easier, thus further reducing the surface roughness. However, for HCP materials like titanium, although the surface temperature of the sample increased more under the influence of impact effect compared to FCC materials, the low-angle grain boundaries did not accumulate at the material surface. Instead, they were minimally created along the boundaries of twinning, as shown in Figure 13f. Obviously, these minimally formed low-angle grain boundaries did not overcome the work hardening induced by the recovery of twinning, resulting in an increase in surface roughness after the impact effect occurred.

## 4. Conclusions

In the present study, a temperature increase induced by ultrasonic energy during the ultrasonic-assisted micro-forging test was obtained successfully and its influence on three metals was investigated. Nevertheless, it should be noted that, although this study has provided a qualitative investigation into surface temperature during the vibration process, the temperature obtained is still an average of the region as close as possible to the surface rather than the instantaneous temperature at the moment of contact between the punch and the material surface. Hence, it is hoped that future research will improve temperature measurement techniques to capture instantaneous temperatures during processing for quantitative studies and will consider additional influencing factors, such as the coefficient of friction.

By processing and analyzing the experimental data and observing the microstructure, several conclusions can be made, as follows:

(1) During ultrasonic-assisted microforging, ultrasonic energy is converted to heat on the surface of the material, causing an increase in temperature, where the average surface temperatures of aluminum, copper, and titanium increase from room temperature to approximately 35°, 41°, and 43°.
(2) During the acoustic softening stage, the increase in temperature is very limited for all three materials, with only a change of almost 2° to 5°.
(3) The rapid rise in surface temperature of each material occurs after the impact effect, and this temperature rise is enhanced by the increase in amplitude, as large amplitudes lead to larger strain rates.
(4) Thermal softening due to the temperature increase caused by the impact effect leads to further softening of the FCC material surface compared to the acoustic softening phase. However, for the HCP material titanium, the impact-effect-induced work hardening has a greater influence than the thermal softening effect.
(5) Microstructure evolutions show that the impact effect on FCC materials could reach deeper into the material due to the temperature diffusion compared to titanium. Also, during the impact effect stage, regenerated twinning, which is reduced under the influence of acoustic softening, consequently results in strain hardening on the surface of titanium.

## Figures and Tables

**Table 1:** Mechanical characteristics of investigated materials.

Material	G (GPa)	E (GPa)	Thermal Conductivity (W/m·K)	$T_{melt}\;(℃)$	Original Ra (μm)
Aluminum	26	69	237	643	5.6
Copper	48	110	401	1083	2.9
Titanium	44	116	21.9	1668	11.4

## Data Availability

The original contributions presented in the study are included in the article, further inquiries can be directed to the corresponding authors.
